# Urine pH and ammonium paediatric reference intervals in first morning spot urine

**DOI:** 10.1515/almed-2025-0070

**Published:** 2025-09-03

**Authors:** Lucas Ramón Díaz-Anadón, Julián Rodríguez, Flor Ángel Ordóñez-Álvarez, Helena Gil-Peña, Óscar David Pons-Belda, Leire Cardo

**Affiliations:** Department of Childhood and Adolescence, 16474 Hospital Universitario Central de Asturias, Oviedo, Spain; Department of Medicine, Universidad de Oviedo, Spain; Department of Paediatrics, Hospital Carmen y Severo Ochoa, Cangas del Narcea, Spain; Paediatric Research Group, Instituto de Investigación Sanitaria del Principado de Asturias (ISPA), Spain; Department of Clinical Analysis, Hospital Can Misses, Eivissa, Spain; Department of Clinical Biochemistry, Laboratory Medicine, 16474 Hospital Universitario Central de Asturias, Oviedo, Spain

**Keywords:** children, reference intervals, single spot urine, urinary ammonium, urine pH

## Abstract

**Objectives:**

Urinary acidification plays a crucial role in acid-base homeostasis, its evaluation being necessary in the differential diagnosis of metabolic acidosis. The practical limitations of the functional tests required to confirm an acidification disorder point out the need for a simple screening method for urinary acidification assessment in spot urine. The best candidates are urine pH and ammonium (NH_4_^+^), but there are few data on their reference values in spot urine in children.

**Methods:**

Our study measured urine pH and NH_4_^+^/creatinine ratio in the first morning fasting urine of 135 healthy children aged between 5 and 14 years. pH was measured by dipstick and potentiometry, concluding that only potentiometric measures were appropriated for assessing urinary acidification in acid-base diseases.

**Results:**

Median urine pH was 5.67 (interquartile range 5.44–6.01), ranging from 4.90 to 7.60. Median NH_4_^+^/creatinine ratio was 4,869 μmol/mmol (interquartile range 3,528–5,918). The reference interval for urine pH ranged from 5.01 to 6.88 and the reference interval for NH_4_^+^/creatinine ratio ranged from 1,646 μmol/mmol to 9,799 μmol/mmol.

**Conclusions:**

These results supply, for the first time, paediatric reference values for both parameters simultaneously, standardizing the sample and method of choice and providing a clinically useful tool for a preliminary evaluation of urinary acidification.

## Introduction

Urinary acidification plays a fundamental role in acid-base homeostasis. Due to its physiological significance and the disorders associated to its impairment (such as incomplete Renal Tubular Acidosis), urinary acidification should be evaluated in some clinical situations. However, the current methods for its evaluation, such as the ammonium chloride oral load or furosemide and fludrocortisone test among others [1], are functional tests whose application in clinical practice is hindered by the possible adverse effects and their tolerability for the patients, especially in paediatrics. Therefore, the need for a simple screening method for urinary acidification disorders is well justified.

The analysis of single spot urine would be the ideal sample to rule out an urinary acidification disorder: a spontaneous urine sample that shows a correct acidification makes the use of a functional test unnecessary [2]. The most important urinary parameters to provide information on urinary acidification status are urine pH and urinary ammonium (NH_4_^+^) evaluated simultaneously, as pH alone is not sufficient to properly assess urinary acidification [1], 3]. However, their application in paediatric clinical practice is still hindered by the paucity of evidence on their reference values in children.

Urine pH follows a circadian rhythm, reaching its minimum before dawn and rising after common Western meals [4], a fact which strongly recommends its evaluation in fasting morning urine samples. Although there are some data published on urine pH values in first morning urine in children [5], there is still no consensus on what type of urine sample should be evaluated: first or second fasting morning urine. Both have drawbacks: The first morning sample might include the theoretically more alkaline urine produced after the previous night’s meal, while the second morning urine is harder to collect, requiring a longer fasting period. The only published study comparing them in adults [6] suggested that the first morning urine pH was a better predictor of a preserved urinary acidification than the second morning sample, but these data have not been replicated yet in children. Furthermore, there is also doubt as to whether dipstick urine pH measurement, the most frequently used method in clinical practice, is accurate enough to be used as a screening test for urinary acidification disorders.

On the other hand, urinary NH_4_^+^ measurement has been historically limited in clinical practice due to technical difficulties associated to traditional measurement methods [7], a fact that has led to the use of indirect methods for estimating urinary NH_4_^+^, such as the urine anion and osmolar gaps, which are far from being accurate [8]. Fortunately, the adaptation of automated plasma NH_4_^+^ quantification methods to measure urine NH_4_^+^ has largely overcome this issue [9] and there are already abundant data on urine NH_4_^+^ excretion in adults in different clinical situations [10], a fact that makes the lack of similar studies in children noteworthy.

The present study aimed to simultaneously evaluate urine pH and NH_4_^+^/creatinine ratio in single spot urine from healthy children between 5 and 14 years of age, in order to establish reference values for both parameters.

## Materials and methods

We prospectively recruited paediatric outpatients who attended the Central University Hospital of Asturias (Oviedo, Spain) from November 2020 to June 2021 for a routine evaluation before scheduled minor surgery and for Allergy Consultations, without concurrent renal or systemic disease (ascertained by a clinical interview). The study was approved by the local Ethics Committee for Investigation with medicinal products and the informed consent of the legal guardians and participants over 12 years of age was obtained. A minimum sample size of 120 was determined, since the parameters’ distributions were unknown beforehand [11].

To solve the question of sample selection, we compared urine pH and NH_4_^+^/creatinine ratio between the first and second fasting morning urine sample in the same day in a subset of children (n=32), with Wilcoxon signed-rank test. The sample of choice was determined accordingly.

Urine samples were collected in non-additive tubes (Vacuette^®^) and delivered to the laboratory within 4 h after collection. Urine pH was measured immediately by potentiometry (pH meter GLP22, Crison^®^ Hach Lange, Berlin, Germany) and by dipstick (Aution sticks 10EA, Arkray; automated reader Aution Max AX-4060, Arkray, Kyoto, Japan). Afterwards, samples were centrifuged (415 × *g*, 5 min) and stored at −80 °C until analysis. Urinary ammonium was measured on a Roche Cobas c501 analyzer (Roche Diagnostics, Mannheim, Germany) by an automated enzymatic plasma ammonium assay, following the protocol validated by Cardo et al. [12]. Creatinine was assayed on a Roche Cobas c701 analyser (Roche Diagnostics, Mannheim, Germany) by Jaffe reaction (CREJ2 creatinine Jaffé Gen.2). Values were subsequently expressed as NH_4_^+^/creatinine ratio.

Statistical analysis was performed using SPSS version 20.0 (SPSS Inc.) and MedCalc version 12.5 (MedCalc Software Ltd). Agreement between pH measurement methods (potentiometry vs. dipstick) was assessed with Cohen’s weighted *kappa* coefficient (by quadratic weights), reclassifying potentiometry values into discrete classes to match dipstick readings (at 0.5 unit intervals). An analysis of the differences between both methods was also performed. The normality of the distributions of the parameters was evaluated using Lilliefors test and non-parametric methods were applied consequently. Data were expressed as median and its interquartile range (IQR), and the 95 % confidence interval (CI). Outliers were identified with Tukey’s test. Differences between gender and correlation with age were evaluated with Mann Whitney U test and Spearman correlation, respectively. 2-sided reference intervals for pH and NH_4_^+^/creatinine ratio were obtained by the non-parametric method (percentiles 2.5 and 97.5), providing the 90 % CI as well for each reference limit [11]. A p-value < 0.05 was considered statistically significant.

## Results and discussion

**Figure 1:**
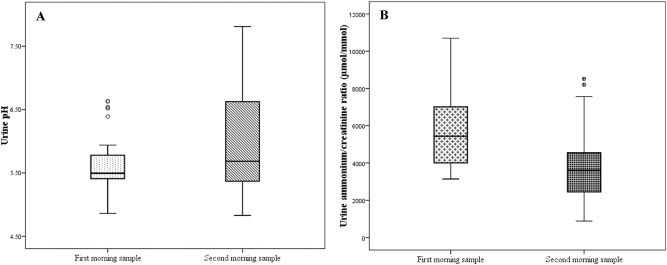
Box plot comparing urine pH (A) and NH_4_^+^/creatinine ratio (B) between first and second morning urine (n=32). Median, 25th and 75th percentiles and outliers are represented.

First and second fasting morning urines were compared in 32 healthy children. Significantly lower pH values (median 5.49 vs. 5.69, p=0.041) with higher NH_4_^+^/creatinine ratios (median 5,438 vs. 3,605 μmol/mmol, p<0.001) were observed in the first morning samples (Figure 1). According to these findings, the first morning urine was considered the sample of choice to evaluate urinary acidification, as lower pH and higher NH_4_^+^/creatinine reference limits may improve their utility as a screening method (Table 1).

**Table 1:** Summary of preanalytical and analytical conditions to evaluate urine pH and NH_4_^+^/creatinine ratio, according to the findings of our study.

Variable	Decision	Justification
Urine sample (1st vs. 2nd)	First morning spot sample	Comparison (n=32) - Lower pH: median 5.49 vs. 5.69 (p=0.041) - Higher NH_4_^+^/creatinine ratio: 5,438 vs. 3,605 μmol/mmol (p<0.001)
pH Measurement method	Potentiometry (pH-meter)	Better resolution Systematic bias of dipstick: - Mean of differences: −0.24 (95 % CI –0.29 to −0.20). - Range of differences: −1.09 to 0.97
NH_4_^+^ Measurement method	Enzymatic assay	Previously validated protocol [12]

Urine samples from 135 children (43 % female) were finally included in the study. Median age of the subjects was 9.17 years (IQR 7.44–11.04). Three minor outliers were found for each parameter but were not excluded, as analytical errors and clinical exclusion criteria were ruled out. Although the agreement in pH measurements by potentiometry (the gold standard method) and by dipstick was moderate (Cohen’s kappa 0.696, 95 % CI 0.628–0.764), the differences between both methods showed a bias; the values by potentiometry were systematically lower: mean of differences −0.24 units (95 % CI –0.29 to −0.20). The differences spanned a whole unit of pH (from a minimum of −1.09 to a maximum of 0.97) (Table 1). Therefore, only potentiometric measurements were considered for this study.

Median urine pH was 5.67 (IQR 5.44–6.01), ranging from 4.90 to 7.60. Median urinary NH_4_^+^/creatinine ratio was 4,869 μmol/mmol (IQR 3,528–5,918), ranging from 1,395 to 12,435 μmol/mmol. There were no significant differences in pH and NH_4_^+^/creatinine ratio between genders and there was no significant correlation of either parameter with age. Thus, reference values were obtained for all children aged 5–14 years. The reference interval for urine pH ranged from 5.01 (90 % CI 4.90–5.13) to 6.88 (90 % CI 6.60–7.60) (Table 2). The reference interval for urinary NH_4_^+^/creatinine ratio ranged from 1,646 μmol/mmol (90 % CI 1,395–2,227) to 9,799 μmol/mmol (90 % CI 8,957–12,435) (Table 2). These data agree with those previously published [5], 13] in different populations, and confirm the first morning urine as the sample of choice for the evaluation of urinary acidification.

**Table 2:** Reference intervals (RI) for urine pH and NH_4_^+^/creatinine ratio in children aged 5–14 years (n=135). CI: confidence interval. IQR: interquartile range.

Parameter, units	RI	90 % CI	Outliers	Median	IQR
pH (−)	5.01–6.88	(4.90–5.13)–(6.60–7.60)	3	5.67	5.44–6.01
NH_4_^+^/creatinine ratio, µmol/mmol	1,646–9,799	(1,395–2,227)–(8,957–12,435)	3	4,869	3,528–5,918

## Conclusions

The present study supplies for the first time paediatric reference intervals for pH and NH_4_^+^/creatinine ratio in first single spot urine from 5 to 14 years, providing a clinically simple and useful tool to preliminarily evaluate urinary acidification when an acidification defect is suspected. More studies are needed to provide reference intervals for younger children.

Furthermore, these findings allow us to establish the urine sample of choice and the best pH measurement method for the detection of urinary acidification defects.
